# Evaluation of the characteristics of leucyl-tRNA synthetase (LeuRS) inhibitor AN3365 in combination with different antibiotic classes

**DOI:** 10.1007/s10096-016-2738-1

**Published:** 2016-08-09

**Authors:** C. G. Monteferrante, A. Jirgensons, V. Varik, V. Hauryliuk, W. H. F. Goessens, J. P. Hays

**Affiliations:** 1 Department of Medical Microbiology and Infectious Diseases, Erasmus University Medical Center Rotterdam (Erasmus MC), Wytemaweg 80, 3015 CN Rotterdam, The Netherlands; 2 Latvian Institute of Organic Synthesis, Riga, Latvia; 3 Institute of Technology, University of Tartu, Nooruse 1, 50411 Tartu, Estonia; 4 Laboratory for Molecular Infection Medicine Sweden (MIMS), Umeå University, Building 6K and 6L, University Hospital Area, 901 87 Umeå, Sweden

## Abstract

Aminoacyl tRNA synthetases are enzymes involved in the key process of coupling an amino acid to its cognate tRNA. AN3365 is a novel antibiotic that specifically targets leucyl-tRNA synthetase, whose development was halted after evaluation in phase II clinical trials owing to the rapid selection of resistance. In an attempt to bring AN3365 back into the developmental pipeline we have evaluated the efficacy of AN3365 in combination with different classes of antibiotic and characterized its mechanism of action. Although we detect no synergy or antagonism in combination with a range of antibiotic classes, a combination of AN3365 with colistin reduces the accumulation of AN3365-resistant and colistin resistance mutations. We also demonstrate that treatment with AN3365 results in the dramatic accumulation of the alarmone (p)ppGpp, the effector of the stringent response—a key player in antibiotic tolerance.

## Introduction

Aminoacyl-tRNA synthetases (aaRS) catalyze an essential step in protein synthesis—the attachment of amino acids to the corresponding tRNAs [1, 2]. They are a promising target for the development of new antibiotic compounds because of their universal nature and the significant structural and biochemical differences between bacterial and eukaryotic aaRS [3, 4]. In this respect, the broad-spectrum antibacterial agent AN3365 was recently developed to selectively target the leucyl-tRNA synthetase (LeuRS) of Gram-negative bacteria [5]. Although the compound has good activity toward drug-resistant Gram-negative organisms, its development was halted during phase II clinical trials owing to the rapid selection of resistant mutants in 3 out of 14 patients enrolled in the study [6].

However, the development of new antibiotics is a slow and costly process and it is therefore crucial that promising lead compounds are extensively tested before they are discarded. Therefore, a further detailed characterization of AN3365 was performed to help guide the further development of more clinically suitable aminoacyl-tRNA synthetase inhibitors for Gram-negative bacteria [7, 8]. The authors evaluated the antibacterial properties of AN3365 using a range of clinically relevant, multi-resistant and susceptible Gram-negative and Gram-positive bacteria: the so-called ESKAPE bacteria, i.e., *Enterococcus faecium* (*E. faecium*), *Staphylococcus aureus* (*S. aureus*), *Klebsiella pneumoniae* (*K. pneumoniae*), *Acinetobacter baumannii* (*A. baumannii*), *Pseudomonas aeruginosa* (*P. aeruginosa*),* Enterobacter* spp., and *Escherichia coli* (*E. coli*). We evaluated the possible synergistic/antagonistic effects of AN3365 in combination with different classes of antibiotics in vitro, and determined the mutation frequencies for the bacterial species* E. coli*, *K. pneumoniae*, and *P. aeruginosa*. Finally, we showed that AN3365 efficiently triggers the so-called bacterial “stringent response” (a stress response activated in bacteria by, for example, amino-acid starvation). Activation of the stringent response is orchestrated by rapid accumulation of the alarmone nucleotide (p)ppGpp, which leads to a reduction in bacterial growth and replication, with an emphasis on the production of proteins involved in amino-acid synthesis instead (https://en.wikipedia.org/wiki/Stringent_response).

## Materials and methods

### Bacterial isolates

To determine the efficacy of AN3365, the in vitro antibacterial activity of AN3365 was tested against a panel of 1,252 bacterial isolates (734 multi-resistant + 518 blood culture isolates) consisting of 889 Gram-negative and 363 Gram-positive bacteria. Most of the strains were obtained from the biobanked strain collection of the Erasmus Medical Center Rotterdam (the Netherlands) with others originating from Iraq, Indonesia, Paraguay, Bangladesh, Saudi Arabia, and Brazil. The multidrug-resistant bacteria included isolates carrying plasmid-encoded pAmpC β-lactamases, extended spectrum beta-lactamases (ESBL), carbapenemase positive (KPC+), oxacillinases-48-like (OXA-48), New Delhi metallo-beta-lactamase (NDM), IMP-type carbapenemases (IMP), Verona integron-encoded metallo-β-lactamase (VIM), methicillin-resistant *Staphylococcus aureus* (MRSA), and vancomycin-resistant enterococci (VanA, VanB). These strains had previously been identified in our laboratory using MALDI-TOF mass spectrometry (Biotyper; Bruker Daltonics, Bremen, Germany), and the presence of the corresponding resistance genes was verified by PCR.

### Susceptibility determination

Minimal inhibitory concentration (MIC) values for different antibiotics were determined using broth microdilution performed in accordance with EUCAST guidelines. Briefly, a standard inoculum of 0.5 McFarland units (approximately 5 × 10^7^ CFU/ml) was prepared in saline for each bacterial isolate. Subsequently, the inoculum was diluted 100 times in Mueller–Hinton II (MH II; Becton, Dickinson and Company) broth to obtain a concentration of approximately 5 × 10^5^ CFU/ml. One hundred microliters of this bacterial suspension was then added to 100 μl of two-fold serial dilutions of the test antibiotic, which had been previously prepared in MH II broth. After incubation at 37 °C, growth or no growth of the bacteria was determined by visually checking the turbidity of the different antibiotic dilutions.

### Synergy testing

In addition to susceptibility testing, we investigated if AN3365 possessed any synergistic properties when used in combination with different classes of clinically relevant antibiotics. Synergy studies were aimed at demonstrating a reduction in the MICs for the combination of antibiotics compared with individual antibiotics used alone. Twenty strains of blood culture isolates of *E. coli* and *K. pneumoniae* were tested for growth in vitro in the presence of combinations of AN3365 with gentamicin (aminoglycoside), cefuroxime (second-generation cephalosporin), ceftazidime (third-generation cephalosporin), meropenem (carbapenem antibiotic), and ciprofloxacin (fluoroquinolone). Synergy testing was performed using the checkerboard methodology as follows: a standard inoculum equal to 0.5 McFarland units was prepared for each bacterial isolate in saline. One hundred microliters of this bacterial suspension was then added to 100 μl of two-fold serial dilutions of the test antibiotics (previously prepared in MH II broth) using a checkerboard pattern—the first antibiotic of the combination was serially diluted along the x-axis, whereas the second antibiotic was diluted along the y-axis. Synergistic or antagonistic activity was determined using the sum of the fractional inhibitory concentration index (ΣFIC). The ΣFIC is calculated as the sum of FIC A + FIC B, where FIC A is the MIC of drug A in the combination of drugs A and B divided by the MIC of drug A alone, plus the MIC of drug B in the combination of drugs A and B divided by the MIC of drug B alone. A combination of drugs is considered synergistic when the ΣFIC is ≤0.5, indifferent when the ΣFIC is >0.5 to <2, and antagonistic when the ΣFIC is ≥2 [9].

### Determination of mutation frequencies

Mutants were obtained by plating dilutions of overnight cultures grown in MH II broth on agar plates containing no antibiotic, and 4-fold or 10-fold MIC concentrations of AN3365, the comparator drug, or combinations of AN3365 and the comparator drug (ceftazidime and colistin). Colonies were counted after 48 h of incubation at 37 °C. The mutation frequency was determined by dividing the number of CFU/ml determined either at 4-fold or 10-fold MIC of AN3365 by the CFU/ml obtained without antibiotic exposure.

### Determination of time-to-mutation

Colonies of *K. pneumoniae* ATCC27853 and *K. pneumoniae* EMC-KPC (a KPC carrying strain of *K. pneumoniae*) were grown to a concentration of 5 × 10^5^ CFU/ml in 96-well plates of: medium consisting of MH II broth; MH II broth containing a sub-inhibitory concentration of AN3365 (0.2 μg/ml); MH II broth containing a sub-inhibitory concentration of colistin (0.06 μg/ml), and MH II broth containing mixed sub-inhibitory concentrations of AN3365 and colistin (0.2 and 0.06 μg/ml). At time points 0, 1, 2, 3, 4, 5, 6, and 24 h, 5 μl of bacterial cultures were transferred to new 96-well plates containing either MH II broth, MH II broth containing 4 times the MIC of AN3365, MH II broth containing 4 times the MIC of colistin, or MH II broth containing 4 times the MIC of both AN3365 and colistin. After overnight incubation at 37 °C, growth/no growth was determined by visually checking the turbidity of the different combinations.

### Determination of (p)ppGpp levels

To determine the accumulation of (p)ppGpp in response to treatment with AN3365, *E. coli* strain MG1655 was grown overnight at 37 °C in morpholinepropanesulfonic acid (MOPS) medium (Teknova) with shaking at 220 rpm. The next day, cells were diluted 100 times in 400 ml MOPS medium and grown at 37 °C until an OD_600_ of 0.4 was achieved. The culture was then split into equal volumes of 200 ml in two flasks, and AN3365 was added to one of these flasks at a concentration 5 times the MIC. After that, cultures were returned to the incubator. Samples for nucleotide measurements were then collected at 15, 30, and 60 min after the addition of AN3365. Nucleotides were then extracted following a protocol adapted from the procedure described by Buckstein and colleagues [10], except that nucleotides were eluted from the HiTrap Q Sepharose FF (GE Healthcare Life Sciences) column using 2 M LiCl in 25 mM Tris pH 8 with a flow rate of 1 ml/min. Elution was followed at 254 nm. Subsequently, 4 volumes of 96 % ethanol (−20 °C) and 4 μl of ice-cold 1 M K_2_HPO_4_ were added to the collected fractions to promote the formation of a nucleotide precipitate and left to precipitate over night at −20 °C. The next day, the sample was centrifuged for 20 min at 5525 G at 4 °C using a swing-out rotor (centrifuge Sigma 4K15C) to collect the precipitate. The supernatant was discarded and the resulting precipitate was washed with 5 ml of 70 % cold ethanol and centrifuged for another 20 min at 5,525 G at 4 °C. After this time, the supernatant was again discarded and the resulting sample was lyophilized and then dissolved in 300 μl of cold mQ with heavy vortexing. Nucleotides were then analyzed by anion exchange high-pressure liquid chromatography (HPLC) using an Agilent system with UV detection at 254 nm and peak height was used for estimating nucleotide amounts. For HPLC, a Waters Spherisorb S5 SAX (4.6 × 250 mm) column equipped with a Spherisorb SAX guard column was used. A flow rate of 1.0 ml/min, and a linear gradient of 100:0 to 0:100 (Buffer A:Buffer B) was used, with a run time of 30 min. Buffer A comprised 0.05 M ammonium phosphate (pH 3.4), and buffer B 0.5 M ammonium phosphate (pH 3.4). Nucleotide standards were obtained from Sigma, and the ppGpp standard was obtained from TriLink BioTechnologies (San Diego, CA, USA).

## Results

### MIC distribution

Determination of MICs using AN3365 and a panel of 1,252 bacterial strains consisting of 889 Gram-negative and 363 Gram-positive bacteria showed that the vast majority of isolates were susceptible to AN3365 at MICs ranging from 0.25 to 2 μg/ml. As previously reported, AN3365 proved to be a very effective drug against Enterobacteriaceae [5], with most of the blood isolates being inhibited at 0.5 μg/ml (52 % of all Gram-negative isolates; Table 1) and the multi-resistant isolates at 1 μg/ml (62 % of all Gram-negative isolates, Table 2). All the Gram-negative strains tested were inhibited at a concentration of 2 μg/ml (Tables 1, 2). For the non-fermenting bacteria, most *P. aeruginosa* isolates were inhibited at 2 μg/ml (69 % of multi-resistant isolates and 56 % of blood culture), whereas *A. baumannii* isolates were mainly inhibited at 1 μg/ml (38 % of multi-resistant isolates and 43 % of blood isolates; Tables 1, 2). In the case of Gram-positive bacteria, *E. faecium* isolates showed MICs ≥128 μg/ml, whereas the growth of MRSA (methicillin-resistant *S. aureus*) and blood isolates of *S. aureus* was inhibited at 4 μg/ml (81 % of MRSA and 92 % of blood isolates). *E. faecalis* isolates were mostly inhibited at 8 μg/ml (60 % of all *E. faecalis* strains). All Gram-positives tested, with the exception of *E. faecium* isolates, were inhibited at a concentration of AN3365 of 16 μg/ml (Tables 1, 2).

**Table 1:** Minimum inhibitory concentrations (MIC) and cumulative MIC distribution for AN3365 using clinical blood culture isolates of Enterobacteriaceae, non-fermentative Gram-negative and Gram-positive bacteria

Isolates	Number of strains (cumulative %) inhibited at MIC (μg/ml) of									Number of isolates tested	MIC	
	0.25	0.5	1	2	4	8	16	32	≥64		50 %	90 %
*A. baumannii*	4 (19)	2 (28)	9 (71)	5 (95)	1 (100)					21	1	2
*C. freundii*		8 (80)	2 (100)							10	0.5	1
*C. koseri*	4 (25)	12 (100)								16	0.5	0.5
*E. cloacae*	1 (2)	39 (82)	7 (96)	2 (100)						49	0.5	1
*E. coli*		33 (65)	18 (100)							51	0.5	1
*E. faecalis*				1 (2)	13 (29)	24 (77)	11 (100)			49	8	16
*E. faecium*									49 (100)	49	128	128
*K. pneumoniae*		34 (69)	11 (92)	4 (100)						49	0.5	1
*M. morganii*	14 (53)	6 (83)	4 (100)							24	0.25	1
*P. aeruginosa*			3 (6)	23 (56)	13 (58)	7 (100)				46	2	8
*P. mirabilis*		14 (29)	32 (95)	2 (100)						48	1	1
*P. vulgaris*		2 (40)	3 (100)							5	1	1
*S. aureus*					48 (92)	3 (98)	1 (100)			52	4	4
*S. marcescens*	4 (8)	40 (80)	6 (100)							50	0.5	1

Bacteria used were clinical isolates originating from the Netherlands, isolated between 2010 and 2014

**Table 2:** The MICs and cumulative MIC distribution for AN3365 in a range of multi-resistant isolates of Enterobacteriaceae, non-fermentative Gram-negative and Gram-positive bacteria

Isolates	Number of strains (cumulative %) inhibited at MIC (μg/ml) of									Number of isolates tested	MIC	
	0.25	0.5	1	2	4	8	16	32	≥64		50 %	90 %
*A. baumannii*		3 (3)	41 (41)	31 (70)	25 (93)	7 (100)				107	2	4
*E. cloacae*	14 (15)	60 (68)	31 (97)	3 (100)						108	0.5	1
*E. coli*		23 (22)	69 (89)	11 (100)						103	1	2
*K. pneumoniae*		30 (27)	68 (82)	14 (100)						112	1	2
*S. aureus*					88 (81)	19 (98)	2 (100)			109	4	8
Enterococci									105 (100)	105	128	128
*P. aeruginosa*			18 (2)	44 (69)	17 (88)	6 (94)	5 (100)			90	2	8

Bacteria used were clinical isolates originating from the Netherlands, Iraq, Indonesia, Paraguay, Bangladesh, Saudi Arabia, and Brazil, isolated between 2010 and 2014

### Synergy studies

No synergistic effect was observed for any of the combinations of AN3365 with different antibiotic classes (Table 3). In combination with other antibiotics, AN3365 mainly showed an indifferent profile, with an FIC 50 % of 1.5 for *K. pneumoniae* and between 1.5 and 2 for *E. coli* and *P. aeruginosa* (Table 3). AN3365 in combination with gentamicin or cefuroxime showed an antagonistic effect for 2 out of the 20 isolates of *K. pneumoniae* tested (data not shown).

**Table 3:** Fractional inhibitory concentration (FIC) range and cumulative FIC for 20 clinical isolates of *E. coli*, *K. pneumoniae*, and *P. aeruginosa* tested for the combination of AN3365 with either gentamicin, cefuroxime, ceftazidime, ciprofloxacin, meropenem or tobramycin

Bacteria		*E. coli*			*K. pneumoniae*			*P. aeruginosa*		
Antibiotics		FIC range	FIC 50 %	95 %	FIC range	FIC 50 %	95 %	FIC range	FIC 50 %	95 %
AN3365	Gentamicin	1.1–2.0	2.0	2.0	0.9–2.4	1.5	2.2	ND	ND	ND
AN3365	Cefuroxime	1.0–2.5	1.5	2.0	1.0–3.0	1.5	3.0	ND	ND	ND
AN3365	Ceftazidime	0.9–2.0	1.5	2.0	1.4–2.5	1.5	2.0	0.7–2.0	1.5	2.0
AN3365	Ciprofloxacin	1.1–2.2	1.5	2.0	1.0–2.5	1.5	2.0	1.2–2.5	2.0	2.0
AN3365	Meropenem	0.7–2.0	2.0	2.0	0.7–2.0	1.4	2.0	1.0–2.5	2.0	2.0
AN3365	Tobramycin	ND	ND	ND	ND	ND	ND	0.7–2.0	1.5	2.0

The combination of AN3365 together with tobramycin for *E. coli* and *K. pneumoniae* was not determined (*ND*) because tobramycin is not clinically relevant for the treatment of *E. coli* or *K. pneumoniae* infections. The combinations of AN3365 together with gentamicin or cefuroxime for *P. aeruginosa* were ND because gentamicin and cefuroxime are not relevant for the treatment of infections caused by *P. aeruginosa*

### Mutation frequencies for AN3365 and colistin

It has been shown that *E. coli*, *P. aeruginosa*, and *K. pneumoniae* possess in vitro mutation frequencies of 1 × 10^−7^ to 8 × 10^−7^ for AN3365 [5]. This mutation rate is high compared with non-antibiotic exposed bacteria, but comparable with that observed when bacteria are exposed to other antibiotics such as ciprofloxacin or ceftazidime [5]. In our experiments, we observed a similar rate of mutation frequencies for at least two strains of *E. coli*, *P. aeruginosa*, and *K. pneumoniae* with mutants appearing with frequencies between 0.76 × 10^−8^ and 2.6 × 10^−7^ (Table 4). When using a combination of AN3365 and colistin, the mutation frequency was much reduced (within the limit of detection of the test protocol), with no mutants appearing when a combination of the two drugs at 4 times their MIC was used (data not shown).

**Table 4:** Mutation frequencies for AN3365 and ceftazidime

Microorganism	Antibiotic	MIC (μg/ml)	Concentration MIC multiple	Mutation frequency
*E. coli* ATCC 25922	AN3365	0.5	4-fold	1.15 × 10^−7^
			10-fold	2 × 10^−8^
*E. coli* MG1655	AN3365	0.5	4-fold	1.5 × 10^−7^
			10-fold	0.76 × 10^−8^
*K. pneumoniae* ATCC 13883	AN3365	1	4-fold	1.4 × 10^−7^
			10-fold	6.6 × 10^−8^
*K. pneumoniae* C10	AN3365	1	4-fold	1.1 × 10^−7^
			10-fold	0.9 × 10^−7^
*P. aeruginosa* ATCC 27853	AN3365	4	4-fold	1.2 × 10^−7^
			10-fold	8.5 × 10^−8^
*P. aeruginosa* JR 326	AN3365	2	4-fold	2.6 × 10^−7^
			10-fold	4.4 × 10^−8^
*E. coli* ATCC 25922	Ceftazidime	0.25	4-fold	ND
			10-fold	ND
*E. coli* MG1655	Ceftazidime	0.5	4-fold	ND
			10-fold	ND
*K. pneumoniae* ATCC 13883	Ceftazidime	0.25	4-fold	ND
			10-fold	ND
*K. pneumoniae* C10	Ceftazidime	>64	4-fold	ND
			10-fold	ND
*P. aeruginosa* ATCC 27853	Ceftazidime	2	4-fold	2.2 × 10^−8^
			10-fold	1.1 × 10^−8^
*P. aeruginosa* JR 326	Ceftazidime	>64	4-fold	ND
			10-fold	ND

Mutation frequencies were determined for *E. coli* ATCC 25922, *E. coli* MG1655, and *K. pneumoniae* ATCC 13883 after overnight culture and assessing the growth or no growth of antibiotic-resistant colonies. *K. pneumoniae* (KPC^+^) C10 and *P. aeruginosa* JR 326 were resistant to ceftazidime

The time taken for resistance mutations to appear when incubated without antibiotic exposure and with sub-inhibitory antibiotic exposure are shown in Table 5. Turbidity, corresponding to the presence of antibiotic resistance mutants, could only be observed in 96-well plates containing single antibiotics, with growth appearing 5–24 h after exposure to the antibiotic (Table 5). When a combination of two antibiotics was used no turbidity was observed. This finding is in agreement with results obtained using solid MH II agar media plates, where mutants for AN3365 and colistin were selected after a single exposure to the individual antibiotic (Table 6). Further, plates containing a combination of AN3365 and colistin showed a marked reduction in mutation frequency even when isolates were grown in medium containing sub-inhibitory concentrations of the two antibiotics for 24 h (Table 6).

**Table 5:** Time to mutation

Time	96-well plate AN3365								96-well plate colistin							
	*K. pneumoniae* ATCC 13883				*K. pneumoniae* EMC-KPC				*K. pneumoniae* ATCC 13883				*K. pneumoniae* EMC-KPC			
	AN3365	Colistin	AN3365+colistin	MHII	AN3365	Colistin	AN3365+colistin	MHII	AN3365	Colistin	AN3365+colistin	MHII	AN3365	Colistin	AN3365+colistin	MHII
0 h	−	−	−	−	−	−	−	−	−	−	−	−	−	−	−	−
1 h	−	−	−	−	−	−	−	−	−	−	−	−	−	−	−	−
2 h	−	−	−	−	−	−	−	−	−	−	−	−	−	−	−	−
3 h	−	−	−	−	−	−	−	−	−	−	−	−	−	−	−	−
4 h	−	−	−	−	−	−	−	−	−	−	−	−	−	−	−	−
5 h	−	−	−	−	−	−	+	−	−	−	−	−	−	−	−	−
6 h	−	−	−	+	−	−	+	−	−	−	−	−	−	+	−	−
24 h	+	−	−	+	+	+	+	−	+	+	+	+	−	+	−	+

The time required for the appearance of *K. pneumoniae* ATCC 13883 and *K. pneumoniae* EMC-KPC mutants resistant to AN3365 or colistin. Bacteria were grown in a medium without antibiotic and with sub-inhibitory concentrations of one or more of the two antibiotics. Cultures were subsequently diluted and inoculated on medium containing no antibiotic, a single antibiotic or a combination of both antibiotics at a concentration 4 times the MIC. When a combination of both antibiotics was used, no change in turbidity, corresponding to an absence of bacterial growth, was observed (data not shown)

**Table 6:** Mutation frequencies for AN3365 and colistin

Microorganism	Antibiotic	MIC (mg/ml)	Concentration MIC multiple	Mutation frequency
*K. pneumoniae* ATCC 13883	AN3365	1	4-fold	1.4 × 10^−7^
			10-fold	6.6 × 10^−8^
*K. pneumoniae* ATCC 13883*	AN3365*	1	4-fold	8.6 × 10^−4^
			10-fold	6.8 × 10^−4^
*K. pneumoniae* ATCC 13883	Colistin	2	4-fold	3.6 × 10^−8^
			10-fold	4 × 10^−9^
*K. pneumoniae* ATCC 13883*	Colistin*	2	4-fold	2.3 × 10^−6^
			10-fold	6.2 × 10^−6^
*K. pneumoniae* ATCC 13883	AN3365 + colistin	1 + 2	4-fold	Not detected
			10-fold	Not detected
*K. pneumoniae* ATCC 13883*	AN3365 + colistin*	1 + 2	4-fold	6.9 × 10^−9^
			10-fold	6.6 × 10^−9^
*K. pneumoniae* EMC-KPC	AN3365	1	4-fold	1.3 × 10^−7^
			10-fold	6 × 10^−8^
*K. pneumoniae* EMC-KPC*	AN3365*	1	4-fold	6.1 × 10^−4^
			10-fold	5.3 × 10^−4^
*K. pneumoniae* EMC-KPC	Colistin	2	4-fold	0.85 × 10^−7^
			10-fold	0.7 × 10^−7^
*K. pneumoniae* EMC-KPC*	Colistin*	2	4-fold	1.8 × 10^−6^
			10-fold	3.1 × 10^−7^
*K. pneumoniae* EMC-KPC	AN3365 + colistin	1 + 2	4-fold	7.5 × 10^−9^
			10-fold	Not detected
*K. pneumoniae* EMC-KPC*	AN3365 + colistin*	1 + 2	4-fold	1.6 × 10^−8^
			10-fold	4.9 × 10^−8^

*K. pneumoniae* ATCC 13883 and *K. pneumoniae* EMC-KPC were grown overnight in MH II medium without antibiotics (*no asterisk*), or with sub-inhibitory concentrations of AN3365 (0.2 mg/ml), colistin (0.06 mg/ml), and AN3365 mixed with colistin (0.2 mg/ml and 0.06 mg/ml; marked with an* asterisk*). The next day, cells were plated on MH II agar plates containing 4-fold or 10-fold MIC concentrations of AN3365, colistin, or a combination of AN3365 and colistin

### Accumulation of (p)ppGpp

**Fig. 1:**
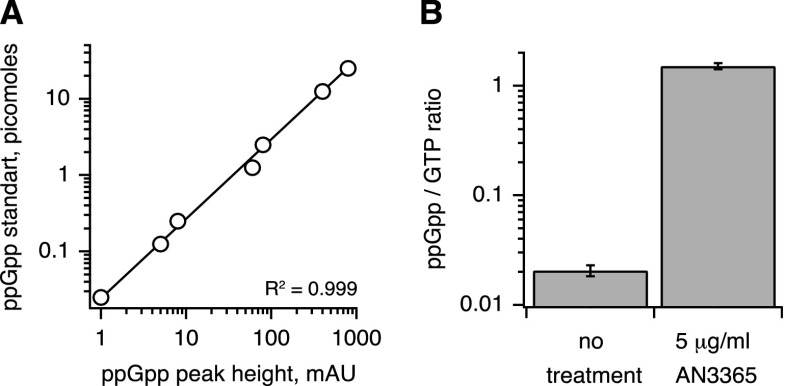
AN3365 induces the accumulation of ppGpp in *E. coli* MG1655. **a** Calibration curve of high-performance liquid chromatography (HPLC) detection of (p)ppGpp shows a linear response of the signal (in mAU) as a function of the amount of injected nucleotide (in picomoles). **b** Liquid culture of *E. coli* MG1655 (MOPS medium supplemented with 0.2 % glucose, OD_600_ = 0.4, 37 °C, shaking at 220 rpm) was treated with 5 μg/ml AN3365 (5xMIC) for 15 min and nucleotides were measured using an HPLC-based approach

AN3365 specifically inhibits *E. coli* LeuRS [5], leading to the accumulation of uncharged tRNA (leading to the induction of the alarmone (p)ppGpp by the stringent response factor RelA [10, 11]). The addition of 5 μg/ml (5xMIC) of AN3365 induced dramatic accumulation of ppGpp, with the ratio of ppGpp to GTP changing from 0.021 ± 0.002 (untreated culture) to 1.51 ± 0.01 after 15 min of AN3365 treatment (Fig. 1.).

## Discussion

Aminoacyl tRNA synthetases may be an excellent target for antibiotics. On the one hand, they catalyze the fundamental process of linking an amino acid to its corresponding tRNA, an essential and universal step for protein synthesis in all life forms [12]. On the other hand, there are significant differences in aminoacyl tRNA synthetases between bacteria and eukaryotes to allow the selective targeting of these enzymes to pathogenic bacteria without interfering with host protein synthesis [1, 12].

In this publication, the authors have re-evaluated AN3365, a novel leucyl-tRNA synthetase inhibitor with a good spectrum of activity toward Enterobacteriaceae, but whose development was previously halted in phase II clinical trials owing to the rapid development of resistance to AN3365 in subjects presenting with complicated urinary tract infections [6]. Our MIC analyses showed that AN3365 was able to efficiently inhibit the growth of multi-resistant, pathogenic, Gram-negative bacteria at concentrations of approximately 1 μg/ml. Comparable MICs were also observed against the non-fermenting bacteria *P. aeruginosa* and *A. baumannii*, and to a lesser extent, activity was observed against the Gram-positive pathogens *S. aureus* and *E. faecalis*. Interestingly, *E. faecium* was not sensitive to the drug at the maximum concentration tested of 128 μg/ml. This is probably because of differences in the structure of the leucyl-tRNA synthetase of this bacterial species that prevent a stable interaction between the inhibitor antibiotic (AN3365) and the *E. faecium* leucyl-tRNA synthetase molecule. To further characterize the activity of AN3365, studies were also performed to discover whether combinations of AN3365 with other classes of antibiotic resulted in a synergistic effect on the antibiotic activity of one or both antibiotics. Unfortunately, however, all of the antibiotic combinations tested showed no synergistic activity.

Mutation frequency experiments using AN3365 showed that exposure to AN3365 led to a relatively high mutation frequency in *E. coli* and *K. pneumoniae* compared with previously published results using the aminoacyl tRNA synthetase inhibitor mupirocin, a tRNA synthetase inhibitor for topical use only, which specifically inhibits the isoleucyl-tRNA synthetase of Gram-positive bacteria [13]. With regard to mupirocin, two resistor phenotypes have been reported: low-level and high-level resistance [14]. Low-level resistance has been linked to single point mutations in the target gene, and high-level resistance is caused by the acquisition of an alternative biosynthetic pathway via a mobile genetic element.

Interestingly, the use of a combination of antibiotics, one bacteriostatic and one bactericidal, i.e., AN3365 and colistin, resulted in a reduction in the mutation frequencies for clinically relevant isolates of *K. pneumoniae*. This finding is probably a result of the inability of bacteria to simultaneously generate multiple viable mutations when exposed to a combination of antibiotics that target two different biological processes within the cell. Perhaps, therefore, it is possible that the use of AN3365 and colistin together (two antibiotics where bacteria can relatively quickly generate resistance to each individual antibiotic via mutation) could enhance the usefulness of these antibiotics in the clinical situation, especially as colistin remains the only current treatment option available for treating extremely resistant Gram-negative infections.

In bacteria, amino acid starvation triggers the accumulation of alarmone nucleotides (p)ppGpp, which in turn, orchestrates the activation of the so-called “stringent response,” a global rewiring of transcription, translation, and metabolism [15]. The stringent response plays an important role in the regulation of bacterial virulence [16] in addition to antibiotic resistance [17] and persistence [18]. Our results indicate that AN3365 strongly induces the stringent response through accumulation of the alarmone (p)ppGpp, which, in turn, generates a change in bacterial metabolism via the down-regulation of the production of ribosomes and the upregulation of, for example, amino-acid synthesis genes. When the stringent response is activated, the bacterial cell enters a “dormant” state [19].

In conclusion, even though the antibiotic AN3365 has been removed from development after disappointing phase II clinical trials, the antibiotic may still have potential clinical value for use in combinatorial antibiotic therapies targeting (multiresistant) bacterial infections, as co-administration of AN3365 with colistin slows down the selection of both AN3365 and colistin-resistant mutants.
